# Phosphodiesterase 5 is main regulator of brown adipocyte differentiation

**DOI:** 10.1186/2050-6511-14-S1-P32

**Published:** 2013-08-29

**Authors:** Ana Kilić, Thorsten Gnad, Linda S Hoffmann, Alexander Pfeifer

**Affiliations:** 1 Institute of Pharmacology and Toxicology, University of Bonn, Sigmund-Freud-Str. 25, Germany

## Background

Obesity, a major threat to global human health, has been shown to be associated with devastating diseases such as stroke, hypertension, cancer and type 2 diabetes. In order to fight the present obesity pandemic it is crucial to better understand regulation of adipocyte differentiation. Phosphodiesterases (PDEs) catalyze hydrolysis of cyclic nucleotides (cyclic adenosine monophosphate (cAMP) and cyclic guanosine monophosphate (cGMP)) to the corresponding 5′ nucleotide monophosphates. To date, eleven different PDEs (PDE1–11) have been characterized, and they differ in their selectivity for cyclic nucleotides, sensitivity to inhibitors and activators, physiological roles, and tissue distribution. The nonselective phosphodiesterase inhibitor isobutylmethylxanthine (IBMX) has been shown to be essential for successful *in vitro* differentiation of adipocytes.

## Results

In the present study, we differentiated brown adipocytes *in vitro* and substituted IBMX with selective PDE-inhibitors; Vinpocetin (PDE1; 10 µM), EHNA (PDE2; 1 µM), Cilostamide (PDE3; 50 nM) and Vardenafil (PDE5; 20 µM) during induction period only or during whole differentiation protocol. Moreover, we directly compared the effects of cAMP and cGMP on brown fat cell differentiation. We show that PDEs (PDE1-5) are expressed in preadipocytes. Omission of IBMX prevented brown adipocyte differentiation. Substitution of IBMX with PDE1, PDE2 and PDE3 inhibitors had no effect on lipid accumulation, while addition of PDE5 inhibitor slightly promoted adipogenesis as seen in Oil Red O staining and expression of adipogenic markers (CCAAT/enhancer-binding protein α (C/EBPα) and ap2). In addition, chronic treatment of brown adipocytes with Vardenafil together with cGMP promoted adipogenesis to the levels reached with IBMX treatment. Interestingly, constant application of 200 µM cGMP promoted lipid accumulation as well as the expression of adipogenic markers (C/EBPα and ap2) while chronic treatment with 200 µM cAMP during brown fat cell differentiation prevented adipogenesis.

## Conclusion

Our *in vitro* results suggest that PDE5 is an important player in brown adipocyte differentiation and indicate that inhibition of PDE5 might have therapeutic implication in treating obesity and related disorders such as stroke and Type 2 diabetes.

